# Pentylamine inhibits humidity detection in insect vectors of human and plant borne pathogens

**DOI:** 10.1038/s41598-022-20488-x

**Published:** 2022-10-06

**Authors:** Iliano V. Coutinho-Abreu, Jonathan Trevorrow Clark, Anandasankar Ray

**Affiliations:** 1 Department of Molecular, Cell and Systems Biology, University of California Riverside, Riverside, CA 92521 USA; 2 Interdepartmental Neuroscience Program, University of California Riverside, Riverside, CA 92521 USA

**Keywords:** Neurophysiology, Olfactory receptors

## Abstract

Insects house humidity-sensing neurons in the antenna, which is presumed to be important for a variety of behaviors and survival since water is a crucial component of the environment. Here we use the simple olfactory system of the Asian Citrus Psyllid (ACP), a citrus pest that transmits a deadly bacterium, to identify volatile amines that significantly inhibited humidity-induced activation of antennal neurons. The inhibition of action potentials is observed by single sensillum recordings and mixing these odorants with humid air abolished the humidity avoidance behavior of ACP. The inhibition is conserved in the humidity-sensing coeloconic neurons of dipteran *Drosophila melanogaster* that are known to detect humidity, but it is not seen in other coeloconic neurons that are not sensitive to humidity. Dipteran mosquitoes *Aedes aegypti* and *Anopheles gambiae* oviposit in water, and the addition of the humidity-inhibiting odorants in a two-choice oviposition assay significantly reduces oviposition. Our results demonstrate that a naturally occurring volatile compound can effectively “mask” detection of an important environmental cue and modify behavior of important vectors of plant and human disease pathogens. Odorants targeting the conserved humidity sensing system of insects, therefore, offer a novel strategy for modifying their behavior.

## Introduction

Humidity represents an important sensory modality for many animal species^1–4^. In addition to acting as a simple cue for detecting water sources, especially critical for small insects prone to desiccation due to large surface area-to-volume ratios^5–7^, humidity serves a diverse range of functions across many species of insects. Foraging hawkmoths use humidity as a means of assessing nectar availability in flowers^8^, and ladybird beetles rely on a range of humidity for tarsal adhesion^9^.

Of particular interest is the role that humidity plays in the behavior of economically important insects, such as vectors of human or agricultural disease-causing pathogens. *Diaphorina citri*, commonly known as the Asian Citrus Psyllid (ACP), is native to China but has spread invasively in North America. The ACP feeds, oviposits, and mates on citrus plants^10^, transmitting to them a bacterial phytopathogen that causes Huanglongbing, and incurable disease of economic significance worldwide^11^. Similarly, the female mosquito, which transmits pathogens that cause millions of disease-related deaths each year, uses humidity in host-seeking behavior^12–14^ and as an oviposition cue^2^. To detect hosts, mosquitoes often rely on complex blends of odorants and CO_2_, the components of which act synergistically by potentiating several attractive olfactory neurons^15^.

In insects, hygroreceptor neurons are usually housed in specific sensilla that contain one of three types of sensory cell: moist-, dry air-, and cold-receptor neurons, yet combinations of chemo- and hygroreceptor neurons housed in the same sensillum have also been described^16^. In the vinegar fly *D. melanogaster*, hygroreceptor neurons are housed in specific coeloconic sensilla^17^ as well as in sensilla located in the sacculus^18,19^. In the mosquito *Aedes aegypti*, an antenna basiconic sensilla detects subtle changes in humidity concentration as well as respond to ammonia and acetone^20^. Similar responses are also observed in the vinegar fly *D. melanogaster* to ammonia and a couple of amines by humidity sensing neurons^17^.

In this study, we investigate the presence of hygroreceptor neurons in the ACP, and the ability of specific chemical volatiles to inhibit water vapor-mediated activation of such neurons in ACP and vinegar flies. We also investigated the potential of chemical volatiles that block the detection of a common environmental cue, such as humidity, and modify humidity-driven behavior of species that shared a common ancestral over 350 million years ago^21^, such as ACP and mosquitoes. The importance of humidity in insect behavior raises the interesting possibility of using “masking” compounds that can block activation of the insect neurons mediating humidity-detection.

## Results

### Amine odorants are antagonists of the humidity sensing neurons in Asian Citrus Psyllid

**Figure 1:**
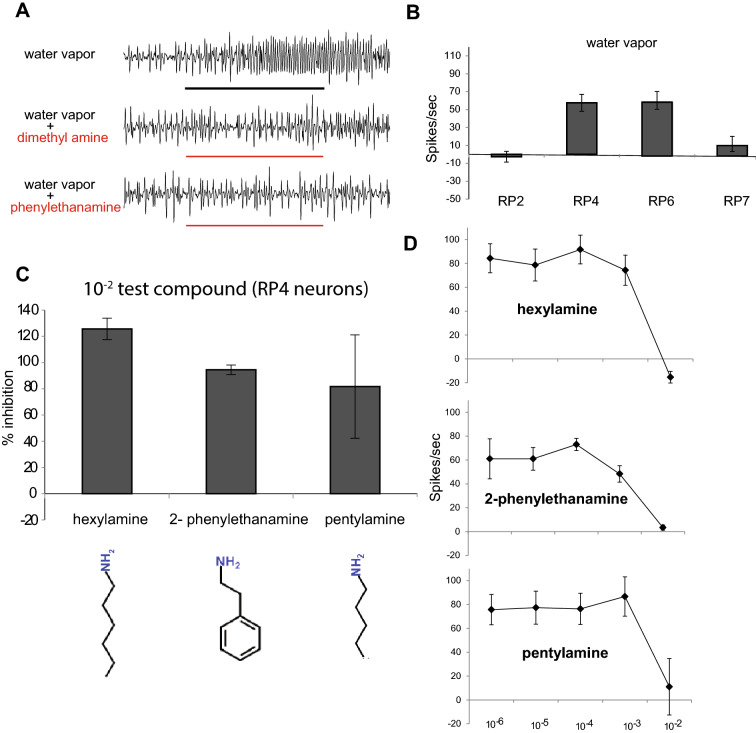
(**A**) Representative traces of neurons from the Asian citrus psyllid (ACP) RP4 sensillum during exposure to a 1-s stimulus of water vapor alone (top), or water vapor coapplied with dimethylamine (middle) and 2-phenylethanamine (bottom). The stimulation period is indicated by the solid bars. Odorant headspace from a 10^–2^ concentration solution was tested for each. (**B**) Mean responses for a 1-s period of stimulation from each ACP RP sensillum in response to water vapor alone. n = 3 sensilla from 3 psyllids. Error bars indicate s.e.m. (**C**) Mean percent inhibition of ACP RP4 neural activity caused by 1% concentration of each of the three displayed compounds. n = 3 sensilla from 3 psyllids. Error bars indicate s.e.m. (**D**) Mean neuronal activity in spikes per second of the ACP RP4 neuron caused by the three displayed compounds across several concentrations. n = 3 sensilla from 3 psyllids. Error bars indicate s.e.m.

The ACP olfactory system is highly specialized yet relatively simple, with peripheral, first-order sensory neurons located on the antennae and housed in 18 trichoid sensilla and 4 pit-like placodea (rhinarial plate [RP]) sensilla: RP2, 4, 6, and 7^22,23^. Each of the four RP sensilla contain three olfactory neurons each, the activities of which have been tested with Single Sensillum Recordings (SSR) against > 100 odorants diluted in paraffin oil as solvent^24,25^. In the present study, we found that water vapor alone increased the activity of at least one neuron in the RP4 and RP6 sensilla, but not of neurons of the RP2 and RP7 sensilla (Fig. 1A and B). As a few odorants appeared to lower the baseline action potential frequency in our previous studies^24,25^, we performed additional SSRs to test if these odorants are antagonists of humidity-sensing sensilla. After confirming that neurons in the RP4 and RP6 neurons are able to detect humidity (Fig. 1B), we coapplied hexylamine, pentylamine, and phenylethanamine each with the water vapor stimulus. All three of these compounds significantly inhibited the humidity-induced activity in these neurons, with 1% hexylamine showing the strongest effect (Fig. 1A, C, D). We also coapplied water vapor with four other amines (pyridine, dimethylamine, N-methyl piperidine, and putrescine hydrochloride) and three acids (2-oxovaleric, hydrochloric, phenylacetic acids), which evoked negligible to weak inhibition of water vapor stimulus (Fig S1).

### Inhibition is conserved in humidity-sensing neurons on the *Drosophila* antenna

**Figure 2:**
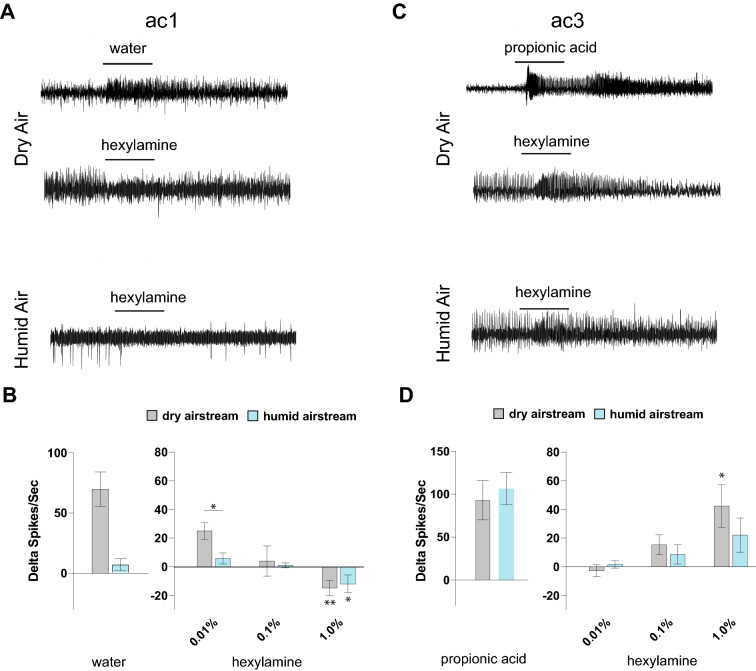
(**A**) Representative traces from the ac1 neurons for a 1-s period of stimulation with water vapor (top) and hexylamine (middle) in a dry air stream, as well as hexylamine (bottom) in a humidified air stream. Odorant headspace from a 10^–2^ concentration solution was tested for each. (**B**) Mean responses. Each count was begun at the start of the increase in spike frequency. All compounds were dissolved in paraffin oil. All recordings were obtained from 3–5 days old wildtype (CS) flies. n = 5–6 sensilla from 5–6 flies. Error bars indicate s.e.m. *p < 0.05; **p < 0.01. (**C**) Representative traces from the ac3 neurons for a 1-s period of stimulation of propionic acid (top) or hexylamine (middle) in a dry air stream, and hexylamine (bottom) in a humidified air stream. Odorant headspace from a 10^–2^ concentration solution was tested for each. (**D**) Mean counts begun at the start of the increase in spike frequency. All recordings were obtained from 3–5 day old wildtype (CS) flies. n = 6 sensilla from 6 flies. Error bars indicate s.e.m. *p < 0.05. All compounds were dissolved in paraffin oil.

In order to test whether the antagonistic effect of the odorants on humidity-sensing neurons in the psyllid antenna was conserved across insect orders^26^, we turned to the well-characterized *D. melanogaster* olfactory system. Antennal olfactory neurons in *Drosophila* that are present in the pit-like sacculus as well as some in the surface coeloconic sensilla have been shown to respond to varying levels of humidity in air and express members of the *Ionotropic receptor* family^17–19,27^. Amongst them the antennal coeloconic sensilla ac1 is sensitive to humidity and are accessible to sensitive SSR recordings^17^. The ac1 neurons express members of the ionotropic receptor family^18,19^, and while they have not been tested for behavioral response to humidity, they show robust electrophysiological response to humidity^17^. We performed SSRs in the ac1 sensillum while it was exposed to varying concentrations of hexylamine in both humidified and dry air backgrounds. While water alone induced a robust increase in ac1 activity (Fig. 2A,B), we found that headspace from 1% hexylamine significantly reduced baseline activity in both the dry and humidified airstreams (Fig. 2A,B). That the inhibitory effect occurs even in a dry airstream suggests that hexylamine acts at some level as an inverse agonist on the ac1 neurons which express *Ionotropic receptors*.

We also performed SSRs in the ac3 sensillum which is not involved in humidity-sensing^17^. We found that hexylamine did not inhibit the baseline activity of these neurons at any concentration tested (Fig. 2C,D), and while minor differences were observed, these differences were not significant at any given concentration between the responses in the humidified vs dry airstream (Fig. 2C,D). Further, 1% hexylamine significantly activated the ac3 neurons. This indicates that the inhibitory effect of hexylamine is specific and not generalizable to all olfactory neurons, or even to those housed in coeloconic sensilla.

### Humidity-mediated behavior of ACP is reduced by antagonist odorants

**Figure 3:**
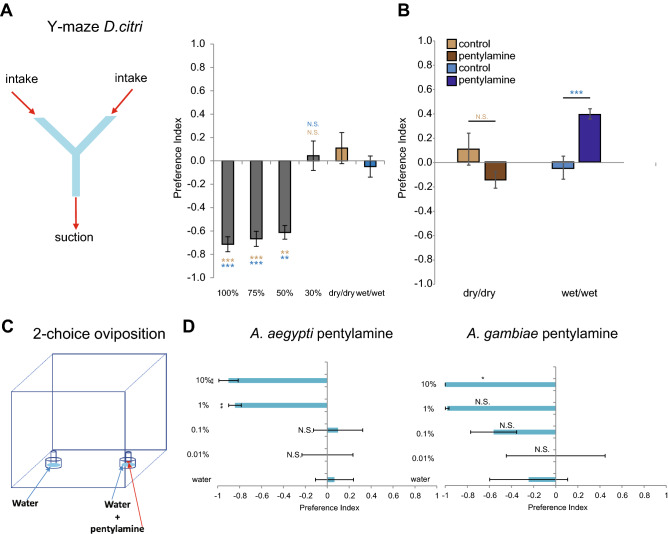
(**A**) Schematic drawing of the Y-maze used for ACP two-choice behavioral assays. Behavioral preference index of ACP to humid air at the indicated concentrations versus dry air, and dry air (dry/dry) or moist air at 75% (wet/wet) pumped into both arms as controls. N = 5–12 trials of 20 male psyllids for each dose. Error bars indicate s.e.m.; ** *p* < 0.01; *** *p* < 0.001. (**B**) Behavioral preference index of ACP to dry air (dry/dry, Left) or moist air at 75% (wet/wet, Right) pumped into both arms as controls and wet or dry air versus wet or dry air with 1% pentylamine. n = 6–12 trials of 20 male psyllids for each humidity level. Error bars indicate s.e.m.; ***p < 0.001. (**C**) Schematic representation of two-choice mosquito oviposition assay. (**D**) Left. Oviposition preference index in *Aedes aegypti* mosquitoes to the indicated concentration of pentylamine versus water alone. n = 4–6 trials with 15 mosquitoes per trial. Error bars represent s.e.m. *p < 0.05. Right. Oviposition preference index in *Anopheles* mosquitoes to the indicated concentration of pentylamine versus water alone. n = 4 and 6 trials respectively, with 15 mosquitoes/trial. Error bars represent s.e.m. *p < 0.05.

To investigate how these compounds may impact the humidity-mediated behavior of ACP, we first tested their behavioral preference for dry air vs air at differing levels of humidity in a Y-maze two-choice assay (Fig. 3A). We found that ACP strongly avoided the arm of the Y-maze when humidity levels reached > 30% (50%, 75%, and 100% moist air), showing preference for the dry-air arm (Fig. 3A). No preference was observed when dry air (dry/dry) or moist air at 75% humidity (wet/wet) was introduced in both arms (Fig. 3A). We then repeated the assay by flowing moist air at 75% humidity in both arms (wet/wet) and yet treated one of the arms with 1% pentylamine (Fig. 3B, right). We found that ACP preferred the side treated with pentylamine, and 70% of the specimens gathered in such a treated arm of the maze (Fig. 3B, right). No significant difference in ACP preference was observed when dry air was pumped in both arms (dry/dry) and 1% pentylamine was applied in one of the arms, suggesting that the odorant by itself imparted no behavior (Fig. 3B, left). These results indicate that pentylamine is able to substantially block humidity-induced aversion in ACP. Pentyamine cannot act as an overriding attraction cue, as no preference was observed for 1% pentylamine in the dry/dry condition (Fig. 3B, left). The simplest interpretation of these results is that in some part the loss of behavior towards humidity is due to inhibition of the detection neurons.

### Humidity-mediated oviposition behavior of mosquitoes is reduced by antagonists

Contrary to avoidance, an example of attractive behavior towards water is seen in gravid female mosquitoes that oviposit in standing water. It is presumed that humidity-sensing could play a critical role in their ability to detect and assess potential oviposition sites^2^ along with other species-specific oviposition pheromones. We were interested in whether a compound that antagonizes humidity-sensing in ACP could also block water-sensing behavior of the mosquito.

We tested oviposition preference in female mosquitoes using a two-choice oviposition assay where the test odorant could be presented as a volatile from a glass vial placed in a water filled petri-dish (Fig. 3C). In the assay with *Aedes* mosquitoes that transmit Dengue and Zika, we found that pentylamine at 1% and 10% drove strong negative preference, and the mosquitoes laid their eggs in the control water dish (Fig. 3D, left). In order to test whether inhibition of oviposition behavior is conserved in the malaria mosquito *A. gambiae,* we repeated the 2-choice oviposition assay with pentylamine. Similar to what was observed in *Aedes*, *Anopheles* females strongly avoided ovipositing on the odorant side at 10% (Fig. 3D, right). Even though *Anopheles* females strongly avoided the oviposition site treated with 1% pentylamine, the results were not statistically significant due likely to the large side bias observed in the control experiments for this species whereby oviposition dishes were treated with no odor (Fig. 3D, right). The simplest interpretation of these results indicates that pentylamine “mask” the humidity as an oviposition cue. However, we cannot rule out that pentylamine might also trigger a repellency pathway in mosquitoes.

## Discussion

Insects inhabit a wide variety of environments with differing levels of humidity. Detecting humidity levels in an insect’s surroundings is important for their response to varying needs for water. Different fruit fly species for example are adapted to live and show preference for relative humidity (RH) specific to their living environment, which ranges from 85% RH in humid rainforests to 20% in dry desert^18^. In this study we used single-sensillum electrophysiology to probe the dangerous pest called the Asian citrus psyllid, which has a numerically simple antenna, and identified neurons that respond to changes in humidity. The ACP feeds, mates, oviposits, and develops on new flush shoots of citrus plants^28,29^. Interestingly, ACP showed preference for the dry arm of the olfactometer over a high-humidity arm in a Y-tube assay. While little is known about ACPs behavioral response to humidity and it is difficult to speculate why they avoid high humidity, a closely related species of insects, pea aphids, are also known to avoid it. The pea aphids sense humidity and heat from the breath of herbivores, and this evokes a behavioral response that prevents them from being eaten: the entire aphid colony drops off the plant^30^. Mosquitoes on the other hand are attracted to humidity near host skin when in a host-seeking state and after a blood meal use humidity sensing to navigate to water bodies as oviposition sites^31^.

Various protein receptors have been proposed to explain humidity (and dryness) sensing^18,19,27,32^. In the model system *D. melanogaster,* some basiconic and coeloconic sensilla on the antennal surface, detect dry air and humidity, and respectively express TRP channels nanchung and water witch^32^. Additionally, the sensilla of the pit-like antennal structure called the sacculus express members of the Ionotropic receptor family, Ir64a and Ir40a, which are required for sensing of humid and dry air, respectively, when associated with the co-receptor Ir25a and the receptor Ir93a^18,19^. These receptors are conserved over hundreds of million years^26,33^, giving support to the possibility that the homologs of such receptors mediate humidity detection in different insect species. Additionally, the Odorant-binding Protein Obp59a is also thought to play a part in humidity sensing^27^.

Using electrophysiology we have identified volatile amines that suppress humidity evoked responses. While the mechanism underlying inhibition is not known, we show that in both species tested, they block humidity-mediated behaviors effectively. In ACP pentylamine reduces the humidity-mediated avoidance, while in mosquitoes it blocks the humidity-mediated attraction to an oviposition site. The mechanism of inhibition may be complex. Ammonia and amines can modify the chemical properties of the air humidity or the temperature of the cuticular surface^16^. Amines might also directly interact with humidity receptors expressed on the dendrites of neurons such as IRs, nanchung and water witch. It has also been reported that insects can express more than one receptor type per neuron^34^, including Ionotropic receptors tuned to amines^35^.

Future studies will shed light upon the mechanism of action of such amines in hygroreceptor neurons housed in different sensilla. Overall, our results indicate that inhibition of the humidity-sensing pathways with specific amines like pentylamine can lead to oviposition deterrence for *Aedes* and *Anopheles* mosquitoes.

## Materials and methods

### Stocks

All ACP experiments were performed inside the Quarantine facility at the University of California, Riverside. ACP (Texas strain) was reared in 40 × 40 × 40 cm cages containing three curry (*Bergera koenegii*) and one citrus (*Citrus volkameriana*) plant (10–15 cm high). Cages were kept at 25 ± 1 °C and 45% relative humidity. Wild type *D. melanogaster* used in the experiments were of the Canton-S strain and reared on standard cornmeal–dextrose media at 22–25 °C. The M form of *A. gambiae* (Herein *Anopheles coluzzi*, Ngousso strain, Cameroon) and *A. aegypti* (Orlando strain) were maintained in a 12:12 (Light:Dark) photocycle at 27 °C and 70% RH.

### Behavior

#### Y-maze

Behavioral experiments for attractiveness and avoidance of ACP to water and odor solutions were carried out in a Y-shaped glass tube (15 mL) with ACP specimens at 4–7 days old and previously starved for 5 h in a humid chamber. Either 100 µL of water alone or odor in water was applied onto a filter paper (Whatman Grade 2) that had been folded onto itself to fit into a glass tubbing. The latter was connected to either side of the Y-shape glass olfactometer. In the assays delivering humidity on one of the arms (or both) of the Y-tube, the filter papers were treated with water or odor in water, which resulted in about 60–70% relative humidity as input in each arm of the olfactometer, as measured before each trial with a handheld hygrometer (Extech EA20 EasyView Hygro-Thermometer). In the trials where dry air was flown in one of the arms (or both) of the Y-tube, a cartridge of silica gel was added in front of the filter paper, reducing the input humidity in the arm to 15–20% even when odor in water was applied onto the filter paper. Each behavioral trial was performed for 10 min at a 55 mL/minute flow rate of charcoal-filtered air. Each treatment was repeated between 5 and 12 times, and the control and treatment arms were alternated between replicates. Replicates displaying less than 30% participation were discarded. At the end of each trial, the number of animals in the control arm, the test arm, and the entry port (ie: non-participants) were counted. Any trial with a participation rate of less than 30% was not included in the analysis.

#### Mosquito oviposition

Oviposition behavior was carried out in bugdorm style (30 × 30 × 30 cm) mosquito cages under 14–10 light cycle, 60% relative humidity, and at 27 °C. Six to seven days after blood feeding, fifteen female mosquitoes were transferred to each bugdorm where two oviposition sites (plastic petri dishes 50 mm) were placed at opposite sides of the cage. The positions of odor-treated and control petri dishes were alternated in the different experimental replicates. In non-contact assays, a glass vial (2 mL total volume) containing 1 mL of either the odor solution or water was placed onto each petri dish, which were filled with 13 mL of autoclaved tap water. A little cup containing sugar solution wicked through a cotton plug was placed between the petri dishes. The experiments were carried out for 24 h. The egg-containing water was drained through Kimwipe tissues and counted under a scope with the help of a cell counting device. The number of eggs found in each oviposition chamber was counted at the end of the experiment.

### Electrophysiology

#### Chemicals

Odorants used in this study were obtained from Sigma-Aldrich at the highest purity available as described previously^24^.

#### Asian citrus psyllid

Single-sensillum recording was performed as previously described^24^ with minor modifications from male ACP (4–7 days old). The odor panel was tested against each type of the four rhinarial plates. Fifty μL of water or odors diluted in water at indicated concentrations were loaded onto cotton wool inserted into Pasteur pipette odor cartridges and used for no more than 4 stimuli each. Odor vapors were delivered from the odor cartridge using a Syntech-55 stimulus controller and diluted approximately three-fold by injecting into a carrier air stream positioned towards the ACP antenna. The baseline activity from one second before the stimulus onset was subtracted from the total activity to obtain a net increase in spikes.

#### *Drosophila*

Adult male flies (*D. melanogaster*) were tested at 3–7 days post-eclosion. Individual flies were inserted into a 1.0 mL pipette tip and secured with molding clay. A glass cover slip was placed underneath the head and a glass pipette was used as a rod to secure an antenna in place. A reference electrode filled with SLR solution was inserted into the eye. Hexylamine was dissolved in paraffin oil to the designated dilutions. A 3 × 35 mm filter paper strip was placed in each 5¾” glass Pasteur pipette to form odor cartridges. Cartridges were allowed to sit and volatilize for 15 min before use. Each cartridge was used no more than twice before being discarded. In the humidified setup, a glass tube was used to maintain a humidified airstream of 10 mL/sec on the preparation, provided by a gas tank routed through a water-filled beaker. The beaker was empty in the dry setup. The odor cartridge was inserted into the side of a glass tube through an opening 130 mm distant from the end of the tube. The end of the tube was placed 5 mm away from the specimen’s antennae, for a total of 135 mm separating the preparation from the point in which odors entered the airstream.

A puff of air was released through the odor cartridge with a Syntech device for 1 s. Spikes were counted during the 1 s application window in the case of owing to a slight delay caused by the increased distance to the antennae. The baseline activity from one second before the stimulus onset was subtracted from the total activity to obtain a net increase in spikes.

### Statistics

Multiple statistical comparisons were performed with non-parametric Kruskal–Wallis test, pairwise comparisons were carried out with the non-parametric Mann–Whitney test for both electrophysiology and behavior data. Preference indexes were calculated as (# in treated arm)—(# in control arm)) / (total # participating) and were ARCSINE transformed for statistical analysis. Statistical comparisons of behavior data were corrected for multiple comparisons with the two stage set up method of Benjamini, Krieger, and Yekutieli and a desired false discovered rate of 0.05.

## Supplementary Information

Supplementary Information 1.

Supplementary Information 2.

## Data Availability

All data generated or analysed during this study are included in this published article [and its supplementary information files].
