# Supplementary material for: Pentylamine inhibits humidity detection in insect vectors of human and plant borne pathogens
Source: Sci Rep. 2022 Oct 6;12:16732. doi: 10.1038/s41598-022-20488-x (PMC9537525; doi:10.1038/s41598-022-20488-x)
Supplement: Supplementary file 1 — Supplementary Information 1. [file 41598_2022_20488_MOESM1_ESM.pdf]

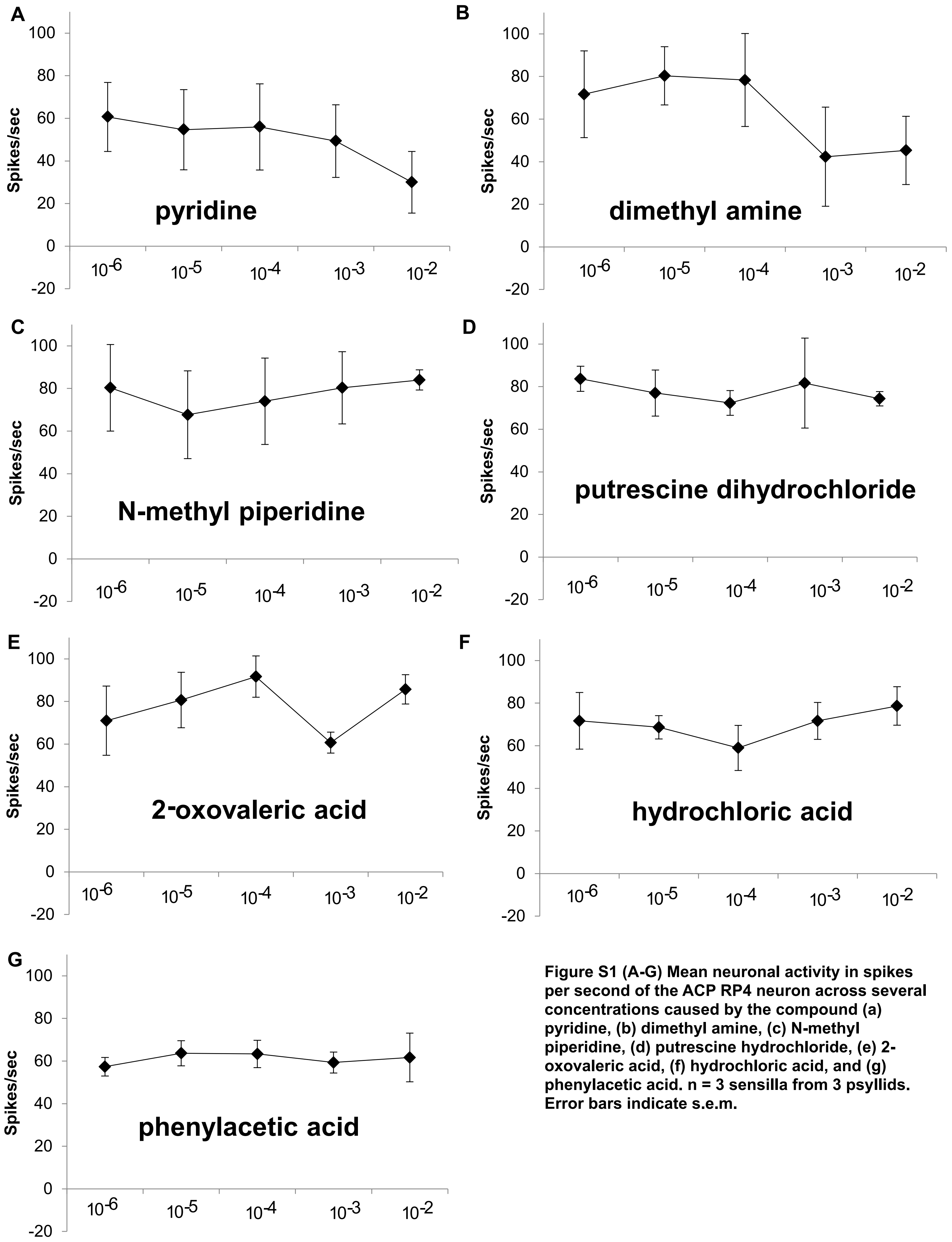

Figure S1 (A-G) Mean neuronal activity in spikes per second of the ACP RP4 neuron across several concentrations caused by the compound (a) pyridine, (b) dimethyl amine, (c) N-methyl piperidine, (d) putrescine hydrochloride, (e) 2-oxovaleric acid, (f) hydrochloric acid, and (g) phenylacetic acid. n = 3 sensilla from 3 psyllids. Error bars indicate s.e.m.
